# Relative transmissibility of shigellosis among different age groups: A modeling study in Hubei Province, China

**DOI:** 10.1371/journal.pntd.0009501

**Published:** 2021-06-10

**Authors:** Zeyu Zhao, Qi Chen, Yao Wang, Meijie Chu, Qingqing Hu, Mikah Ngwanguong Hannah, Jia Rui, Xingchun Liu, Yunhan Yu, Fuwei Zhao, Zhengyun Ren, Shanshan Yu, Ran An, Lili Pan, Yi-Chen Chiang, Benhua Zhao, Yanhua Su, Bin Zhao, Tianmu Chen

**Affiliations:** 1 State Key Laboratory of Molecular Vaccinology and Molecular Diagnostics, School of Public Health, Xiamen University, Xiamen City, Fujian Province, People’s Republic of China; 2 Hubei Provincial Center for Disease Control and Prevention, Wuhan City, Hubei Province, People’s Republic of China; 3 Division of Public Health, School of Medicine, University of Utah, Presidents Circle, Salt Lake City, Utah, United States of America; 4 Medical College, Xiamen University, Xiamen City, Fujian Province, People’s Republic of China; 5 School of Statistics, Beijing Normal University, Beijing City, People’s Republic of China; 6 Medical Insurance Office, Xiang’an Hospital of Xiamen University, Xiamen City, Fujian Province, People’s Republic of China; The Hospital for Sick Children, CANADA

## Abstract

Shigellosis is a heavy disease burden in China especially in children aged under 5 years. However, the age-related factors involved in transmission of shigellosis are unclear. An age-specific Susceptible–Exposed–Infectious/Asymptomatic–Recovered (SEIAR) model was applied to shigellosis surveillance data maintained by Hubei Province Centers for Disease Control and Prevention from 2005 to 2017. The individuals were divided into four age groups (≤ 5 years, 6–24 years, 25–59 years, and ≥ 60 years). The effective reproduction number (*R*_eff_), including infectivity (*R*_I_) and susceptibility (*R*_S_) was calculated to assess the transmissibility of different age groups. From 2005 to 2017, 130,768 shigellosis cases were reported in Hubei Province. The SEIAR model fitted well with the reported data (*P* < 0.001). The highest transmissibility (*R*_eff_) was from ≤ 5 years to the 25–59 years (mean: 0.76, 95% confidence interval [CI]: 0.34–1.17), followed by from the 6–24 years to the 25–59 years (mean: 0.69, 95% CI: 0.35–1.02), from the ≥ 60 years to the 25–59 years (mean: 0.58, 95% CI: 0.29–0.86), and from the 25–59 years to 25–59 years (mean: 0.50, 95% CI: 0.21–0.78). The highest infectivity was in ≤ 5 years (*R*_I_ = 1.71), and was most commonly transmitted to the 25–59 years (45.11%). The highest susceptibility was in the 25–59 years (*R*_S_ = 2.51), and their most common source was the ≤ 5 years (30.15%). Furthermore, “knock out” simulation predicted the greatest reduction in the number of cases occurred by when cutting off transmission routes among ≤ 5 years and from 25–59 years to ≤ 5 years. Transmission in ≤ 5 years occurred mainly within the group, but infections were most commonly introduced by individuals in the 25–59 years. Infectivity was highest in the ≤ 5 years and susceptibility was highest in the 25–59 years. Interventions to stop transmission should be directed at these age groups.

## Author summary

Shigellosis has a heavy disease burden in developing countries. The incidence of shigellosis is highest in children aged under 5 years. The transmission characteristics of shigellosis are unclear in different groups, such as age and occupation groups. In this study, we applied an age-specific mathematical model to analyze data on shigellosis built in Hubei Province from 2005 to 2017. The study found that: 1) Shigellosis transmission in children aged 5 years and under occurred among themselves, but was most commonly transmitted by adults aged 25–59 years; 2) The transmission interaction was greatest between children aged 5 years and under, and adults aged 25–59 years; 3) Infectivity was highest in children aged 5 years and under, and susceptibility was highest in adults aged 25–59 years.

## Introduction

Shigellosis, an intestinal infectious disease caused by *Shigella* spp, is common in children aged under 5 years in low- and middle-income countries, and usually leads to acute infectious diarrhea [1]. *Shigella* is the second most common cause of diarrheal deaths worldwide. Despite the decline in mortality due to diarrhea, its incidence remains high, particularly in developing countries [2,3]. According to a report by the Chinese Center for Disease Control and Prevention (CDC), from 2005 to 2010, there were 250,000–500,000 cases of shigellosis annually [4].

Shigellosis causes a heavy disease burden among children under the age of 5 in developing countries [5–7]. The annual number of shigellosis cases in developing countries is estimated to be 113,163,260; 14,654,230; 30,065,470; 5,296,565 among those aged under 5 years, 5–14 years, 15–59 years, and 60 years and over, respectively [3]. Furthermore, the incidence of shigellosis differs by age, and is highest in children aged under 5 years [8–10]. The different disease burden also varies by age. The incidence of shigellosis is directly related to a low level of hygiene [11]. The differences in incidence by age may be related to age-related differences in lifestyle. Shigellosis is primarily transmitted from person-to-person [1,12]; therefore, interpersonal transmission, particularly among different age groups, should be considered.

Several studies of shigellosis have used the autoregressive integrated moving average (ARIMA) model [13–15]. Bai et al. [16] developed a susceptible–infectious–recovered–susceptible (SIRS) model of shigellosis with seasonal fluctuations in 2011, but did not explore the transmission route from water/food-to-person. Another study applied the Susceptible–Exposed–Infectious/Asymptomatic–Recovered–Water/Food (SEIARW) model to discuss features of a shigellosis outbreak in a school, but did not explore age-specific transmission [17]. Furthermore, several studies have found that the water/food-to-person route does not account for shigellosis transmission [1,18,19]. Therefore, we simplified the SEIARW model and built an age-specific Susceptible–Exposed–Infectious/Asymptomatic–Recovered (SEIAR) model to explore the characteristics of interpersonal transmission. We adopted the effective reproduction number (*R*_eff_), the average number of secondary cases per infectious case in a population made up of both susceptible and non-susceptible hosts; infectivity (*R*_I_), the ability of a pathogen to establish an infection; and susceptibility (*R*_S_), lack of ability to resist a pathogen, to assess the transmissibility of shigellosis among different age groups.

In this study, an age-specific SEIAR model was used to describe the transmission of shigellosis in Hubei Province, China, and to quantify the transmissibility of shigellosis among different age groups.

## Methods

### Ethics statement

Disease surveillance and investigation is part of the mandate of the CDC in Hubei Province; therefore, the study was exempted from ethics review and the requirement for informed consent was waived by the Medical Ethics Committee of Hubei Center for Disease Control and Prevention on the following grounds: (1) all data analyzed were anonymized; (2) neither medical intervention nor biological samples were involved; and (3) study procedures and results did not affect the clinical management of patients.

### Study design

**Fig 1:**
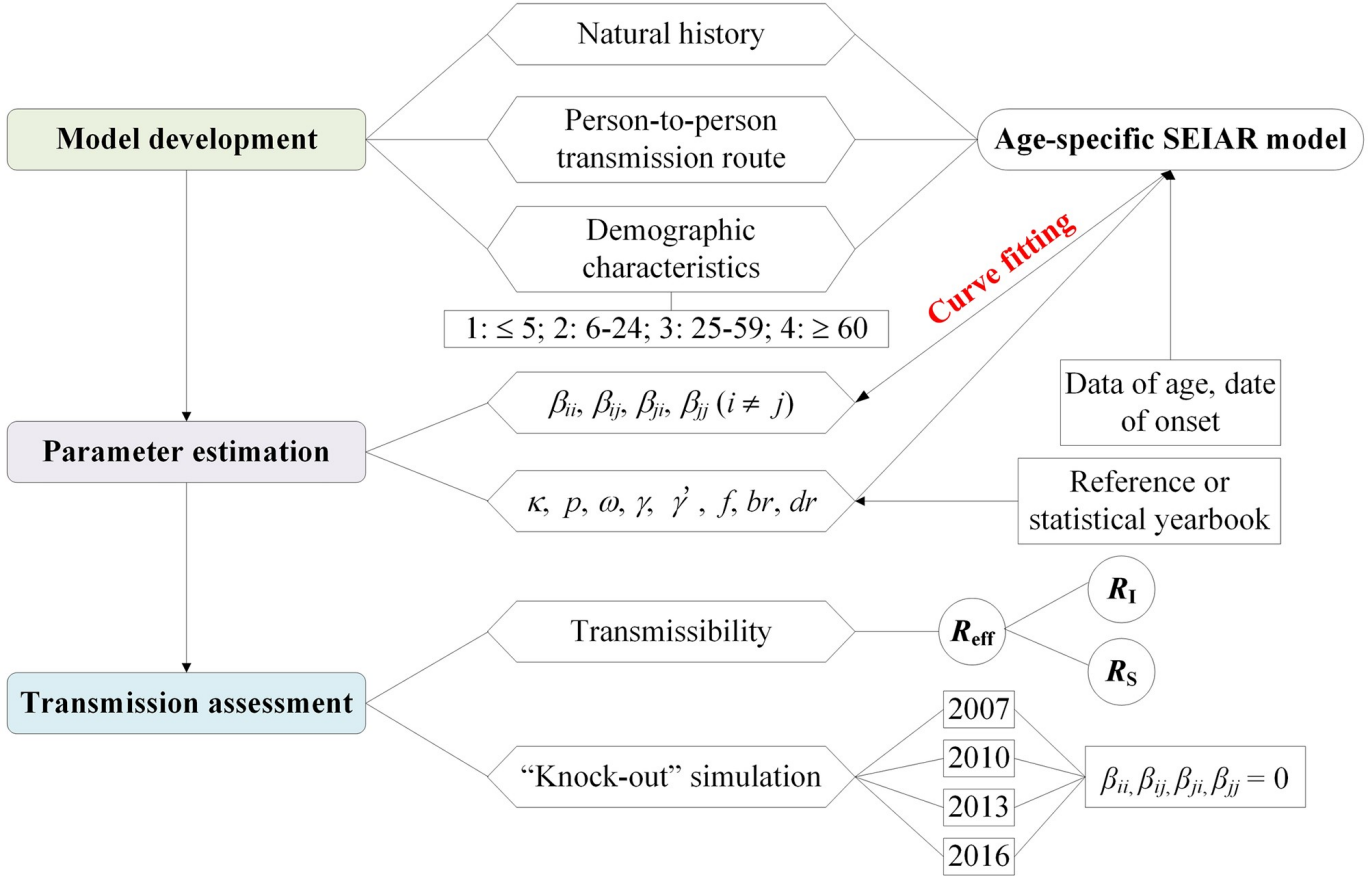
Flowchart of study design from model development to transmission assessment.

An age-specific SEIAR model was built according to the different incidence in the four age groups and the natural history of shigellosis. Parameters were estimated, and the indictor (*R*_eff_) was calculated (Fig 1). In China, preschool children are mainly aged ≤ 5 years, and preschool children have different behaviors and contacts to students (mainly aged 6–24 years). Furthermore, there is different lifestyle among preschool children, students, workers (mainly aged 25–59 years) and older adults (aged ≥ 60 years). We divided the total population into four age groups and used the subscripts *i* and *j* to represent age group 1 to 4 (*i* ≠ *j*; 1: ≤ 5 years; 2: 6–24 years; 3: 25–59 years; and 4: ≥ 60 years).

### Data sources

We collected data (including sex, age, occupation, address, date of onset, date of diagnosis, etc.) of reported cases built from a surveillance of shigellosis in Hubei Province from January 1, 2005, to December 31, 2017. In this study, people were divided into four age groups (≤ 5 years, 6–24 years, 25–59 years, and ≥ 60 years) and the number of reported cases per day were recorded. Meanwhile, the birth rate, death rate, and total population of Hubei Province (including Wuhan City, Huangshi City, Shiyan City, Yichang City, Xiangyang City, Ezhou City, Jinmen City, Xiaogan City, Jinzhou City, Huanggang City, Xianning City, Suizhou City, Enshi City, Xiantao City, QianJiang City, Tianmen City and Shennongjia Forest Area) from 2005 to 2017 were obtained from the Hubei Statistical Yearbook.

### Shigellosis model among different age groups

**Fig 2:**
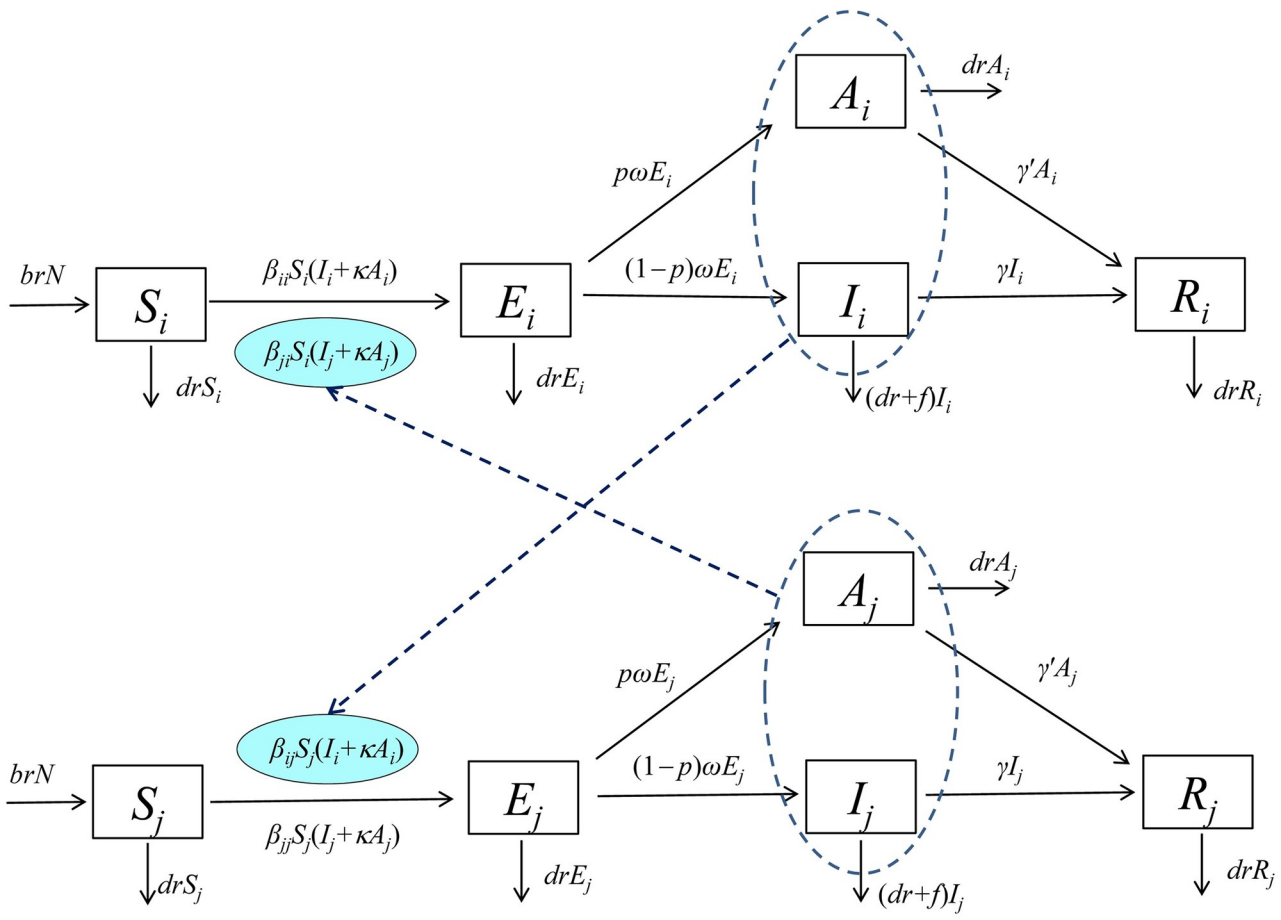
Flowchart of age-specific SEIAR model of shigellosis. *i* ≠ *j*; *i* and *j* represent age group ≤ 5 years old, 6–24 years old, 25–59 years old, and ≥ 60 years old, respectively.

In contrast to Pitzer’s research [20], used seasonal age-structured SIR model to explore the relationship between the number of cases and the average age of first infection, our previous studies adopted two sub-models to describe the transmission interaction in different sex and age groups [19,21]. In the model (Fig 2), the routes of transmission were from person-to-person in the four age groups. We defined susceptible (*S*), exposed (*E*), infectious (*I*), asymptomatic (*A*) and recovered (*R*) individuals (Table 1). An age-specific SEIAR model was developed based on the following conditions:

1. Susceptible individuals of different age groups were infected by contact with symptomatic/asymptomatic people;
2. Shigellosis could be transmitted within an age group. The relative rate of transmission among age groups *i* and *j* were *β*_*ii*_ and *β*_*jj*_ respectively.
3. Shigellosis could be transmitted between different age groups. The relative rate of transmission from age group *i* to *j* was *β*_*ij*_ and from age group *j* to *i* is *β*_*ji*_.

**Table 1:** Variables with the age groups transmission SEIAR model.

Variable	Description	Unit
*S*_*i*_	Susceptible individuals density of age group *i*	Individuals·km^-2^
*S*_*j*_	Susceptible individuals density of age group *j*	Individuals·km^-2^
*E*_*i*_	Exposed individuals density of age group *i*	Individuals·km^-2^
*E*_*j*_	Exposed individuals density of age group *j*	Individuals·km^-2^
*I*_*i*_	Infectious individuals density age group *i*	Individuals·km^-2^
*I*_*j*_	Infectious individuals density age group *j*	Individuals·km^-2^
*A*_*i*_	Asymptomatic individuals density age group *i*	Individuals·km^-2^
*A*_*j*_	Asymptomatic individuals density age group *j*	Individuals·km^-2^
*R*_*i*_	Recovered individuals density age group *i*	Individuals·km^-2^
*R*_*j*_	Recovered individuals density age group *j*	Individuals·km^-2^
*N*	Total number of population density	Individuals·km^-2^

The following conditions were applied:

1. Shigellosis was not transmitted vertically, and the individuals born in age group 1 were all susceptible. The natural birth rate was *br*, and the natural mortality rate was *dr*;
2. The incubation period of the exposed population was 1/*ω*. Therefore, the rates of change from *E* to *A* and *E* to *I* were equal. We defined *p* (0 ≤ *p* ≤ 1) as the proportion of asymptomatic infections. Exposed individuals would become asymptomatic person *A* with a daily rate of *pE*, and become symptomatic at a rate of (1-*p*)*E*;
3. Individuals *I* and *A* would become recovered person (*R*) after an infectious of 1/*γ* and 1/*γ’*;

The model was expressed as follows:

i≠j

$$\frac{dS_{i}}{dt}=brN-\beta_{ii}S_{i}\left(I_{i}+\kappa A_{i}\right)-\beta_{ji}S_{i}\left(I_{j}+\kappa A_{j}\right)-drS_{i}$$

$$\frac{dE_{i}}{dt}=\beta_{ii}S_{i}\left(I_{i}+\kappa A_{i}\right)+\beta_{ji}S_{i}\left(I_{j}+\kappa A_{j}\right)-\omega E_{i}-drE_{i}$$

$$\frac{dI_{i}}{dt}=\left(1-p\right)\omega E_{i}-\gamma I_{i}-(drI_{i}+fI_{i})$$

$$\frac{dA_{i}}{dt}=p\omega E_{i}-\gamma^{'}A_{i}-drA_{i}$$

$$\frac{dR_{i}}{dt}={\gamma I}_{i}+{\gamma^{'}A}_{i}-drR_{i}$$

$$\frac{dS_{j}}{dt}=brN-\beta_{jj}S_{j}\left(I_{j}+\kappa A_{j}\right)-\beta_{ji}S_{j}\left(I_{i}+\kappa A_{i}\right)-drS_{j}$$

$$\frac{dE_{j}}{dt}=\beta_{jj}S_{j}\left(I_{j}+\kappa A_{j}\right)+\beta_{ji}S_{j}\left(I_{i}+\kappa A_{i}\right)-\omega E_{fj}-drE_{j}$$

$$\frac{dI_{j}}{dt}=\left(1-p\right)\omega E_{j}-\gamma I_{j}-(drI_{j}+fI_{j})$$

$$\frac{dA_{j}}{dt}=p\omega E_{j}-\gamma^{'}A_{j}-drA_{j}$$

$$\frac{dR_{j}}{dt}={\gamma I}_{j}+{\gamma^{'}A}_{j}-drR_{j}$$

$$N=S_{i}+E_{i}+I_{i}+A_{i}+R_{i}$$

The left side of the equation shows the instantaneous rate of change of *S*, *E*, *I*, *A* and *R* at time *t*. In the model, the transmissibility estimated by the effective reproduction number (*R*_eff_), the average number of secondary cases per infectious case in a population made up of both susceptible and non-susceptible individuals, was calculated as follows:

$$R_{eff}=\beta N(\frac{1-p}{\gamma }+\frac{\kappa p}{\gamma^{'}})$$

Furthermore, the infectivity (*R*_I_), the ability of a pathogen to establish an infection and susceptibility (*R*_S_), lack of ability to resist a pathogen, of shigellosis were calculated using the following equations:

$$R_{I-i}=\sum_{j=1}^{n}R_{eff-ij}$$

$$R_{S-i}=\sum_{j=1}^{n}R_{eff-ji}$$

In the above equations, *n* = 4. For example, *R*_I_ of age group 1 is the sum of *R*_eff-11_, *R*_eff-12_, *R*_eff-13_ and *R*_eff-14_, and *R*_S_ of age group 1 is the sum of *R*_eff-11_, *R*_eff-21_, *R*_eff-31_, and *R*_eff-41_.

To further quantify the contribution of different transmission routes, including 16 parameters of *β* that fit by the SEIAR model, the “knock-out” simulation method which is theoretically derived from the method of gene “knock-out” (a genetic technique in which one of an organism’s genes is made inoperative) was employed. In this study, a “knock-out” simulation (in which different routes of shigellosis transmission among various age groups, respectively) was according to three sets of scenarios: A) scenario I: including a control and sixteen sub-scenarios (*β*_*ij*_ = 0); B) scenario II: including a control and eleven sub-scenarios (*β*_I_ to *β*_X_); C) scenario III: including a control and five sub-scenarios (*β*_I_ to *β*_IV_).

### Estimation of parameters

Water/food transmission may still play a significant role in transmission events that are often further propagated by person-to-person transmission [1]. Combining with our previous model studies [18,19], the transmission route from water/food-to-person of shigellosis had already been cutting off. Therefore, we only considered the person-to-person transmission. The values *κ*, *ω*, *γ*, and *γ*’ were set to 0.3125, 1.0000, 0.0741, and 0.0286, respectively, according to our previous results [17]. Setting *p* = 0.1 the proportion of asymptomatic individuals ranged from 0.0037 to 0.2700 [22–24]. The shigellosis fatality rate in China decreased from 0.00088 per year to 0.00031 per year from 1991 to 2000 [25]. Considering that the fatality rate of shigellosis was extremely low, we set *f* = 0. As only age ≤ 5 years had a birth population, *br* was considered a conditional parameter in the model. When *i*, *j* = 1, the value of *br* was obtained from the Hubei Statistical Yearbook. While *i*, *j* ≠ 1, we set *br* = 0. The values of *β*_*ij*_ were calculated by fitting the curve of the model. The description and source of the parameters are shown in Table 2.

**Table 2:** Parameter description and values of SEIAR model.

Parameter	Description	Unit	Value	Range	Method
*β*_*ii*_ *	Transmission relative rate among age group *i*	Individuals^-1^·days^-1^	-	≥ 0	Curve fitting
*β*_*ij*_ *	Transmission relative rate from age group *i* to *j*	Individuals^-1^·days^-1^	-	≥ 0	Curve fitting
*β*_*ji*_ *	Transmission relative rate from age group *j* to *i*	Individuals^-1^·days^-1^	-	≥ 0	Curve fitting
*β*_*jj*_ *	Transmission relative rate among age group *i*	Individuals^-1^·days^-1^	-	≥ 0	Curve fitting
*κ*	Relative transmissibility rate of asymptomatic to symptomatic individuals	1	0.3125	0–1	References [6]
*p*	Proportion of the asymptomatic	1	0.1	0.0037–0.27	References [20–22]
*ω*	Incubation relative rate	days^-1^	1	≥ 0	References [6]
*γ*	Recovery rate of the infectious	days^-1^	0.0741	≥ 0	References [6]
*γ’*	Recovery rate of the asymptomatic	days^-1^	0.0286	≥ 0	References [6]
*f*	Fatality of the disease	1	0	0–1	References [19]
*br*	Birth rate of the population	1	-	0.0087–0.0126	Hubei Statistical Yearbook
*dr*	Death rate of the population	1	-	0.0057–0.0070	Hubei Statistical Yearbook

*: *i* and *j* represent age group 1 to 4, respectively; *i* ≠ *j*

According to reported incidence of shigellosis from 2005 to 2017 in Hubei Province, we divided the year into four stages representing different epidemics (S1 Fig). The“knock-out” simulation was employed in 2007, 2010, 2013 and 2016 which were chosen from four stages: Stage 1 was from 2005 to 2008; Stage 2 was from 2009 to 2011; Stage 3 was from 2012 to 2014; Stage 4 was from 2015 to 2017.

### Reinfection analysis

We assumed that recovered individuals could become susceptible individuals with a rate of *x*. The flowchart of the model shown in S2 Fig. In the model, the equations of compartments *S* and *R* were changed as follows:

$$\frac{dS_{i}}{dt}=brN-\beta_{ii}S_{i}\left(I_{i}+\kappa A_{i}\right)-\beta_{ji}S_{i}\left(I_{j}+\kappa A_{j}\right)-drS_{i}+xS_{i}$$

$$\frac{dR_{i}}{dt}={\gamma I}_{i}+{\gamma^{'}A}_{i}-drR_{i}-xS_{i}$$

$$\frac{dS_{j}}{dt}=brN-\beta_{jj}S_{j}\left(I_{j}+\kappa A_{j}\right)-\beta_{ji}S_{j}\left(I_{i}+\kappa A_{i}\right)-drS_{j}+xS_{j}$$

$$\frac{dR_{j}}{dt}={\gamma I}_{j}+{\gamma^{'}A}_{j}-drR_{j}-xS_{j}$$

In this study, we set *x* to 0.0, 0.1, 0.5 and 1.0, to re-calibrate the curve and compare the *R*_eff_. The data from six parts in 2005 were used for the reinfection analysis.

### Simulation method and statistical analysis

The annual data were divided into multiple parts and simulated respectively using Berkeley Madonna 8.3.18 (Department of Molecular and Cellular Biology, University of California, Berkeley, CA, USA, http://www.berkeleymadonna.com). The simulation methods (Runge–Kutta method of order four with tolerance set to 0.001) were the same as those used in previously published research [26–29]. Berkeley Madonna adopted curve fitting for the least root-mean-square deviation. The annual data were divided into multiple parts and the simulated time-step was one day; for example, the data from 2005 were divided into 22 parts (S3 Fig). Microsoft Office Excel 2019 (Microsoft, Redmond, WA, USA) and GraphPad Prism 7.0 (GraphPad Software, La Jolla, CA) were used for figure development and data analysis. The coefficient of determination (*R*^2^) and Student’s *t* test were calculated using by SPSS 21.0 (IBM Corp, Armonk, NY, USA) to judge the goodness of fit.

Student’s *t* test was also used to compare the difference of the two simulation methods about initial value setting in 2005, which were as follows:

#### Method 1

The other initial values were kept unchanged (*S* = total population of each year, *E* = 0, *A* = 0, *R* = 0), and we set the initial values of *I* to the value at the end of the previous part;

#### Method 2

The initial values of *S*, *E*, *I*, *A* and *R* were set to the value at the end of the previous parts.

### Sensitivity analysis

In our model, the five parameters including *κ* (0–1), *p* (0.0037–0.2700), *ω* (0.3333–1), *γ* (0.0477–0.1428) and *γ*’ (0–0.0357) were split into 1,000 values according to their range. The mean and mean ± standard deviation (SD) were calculated after the sensitivity analysis of the model. As the simulation method was the same for each year, the sensitivity analysis was performed using the 2005 data.

## Results

### Epidemiological characteristics

**Fig 3:**
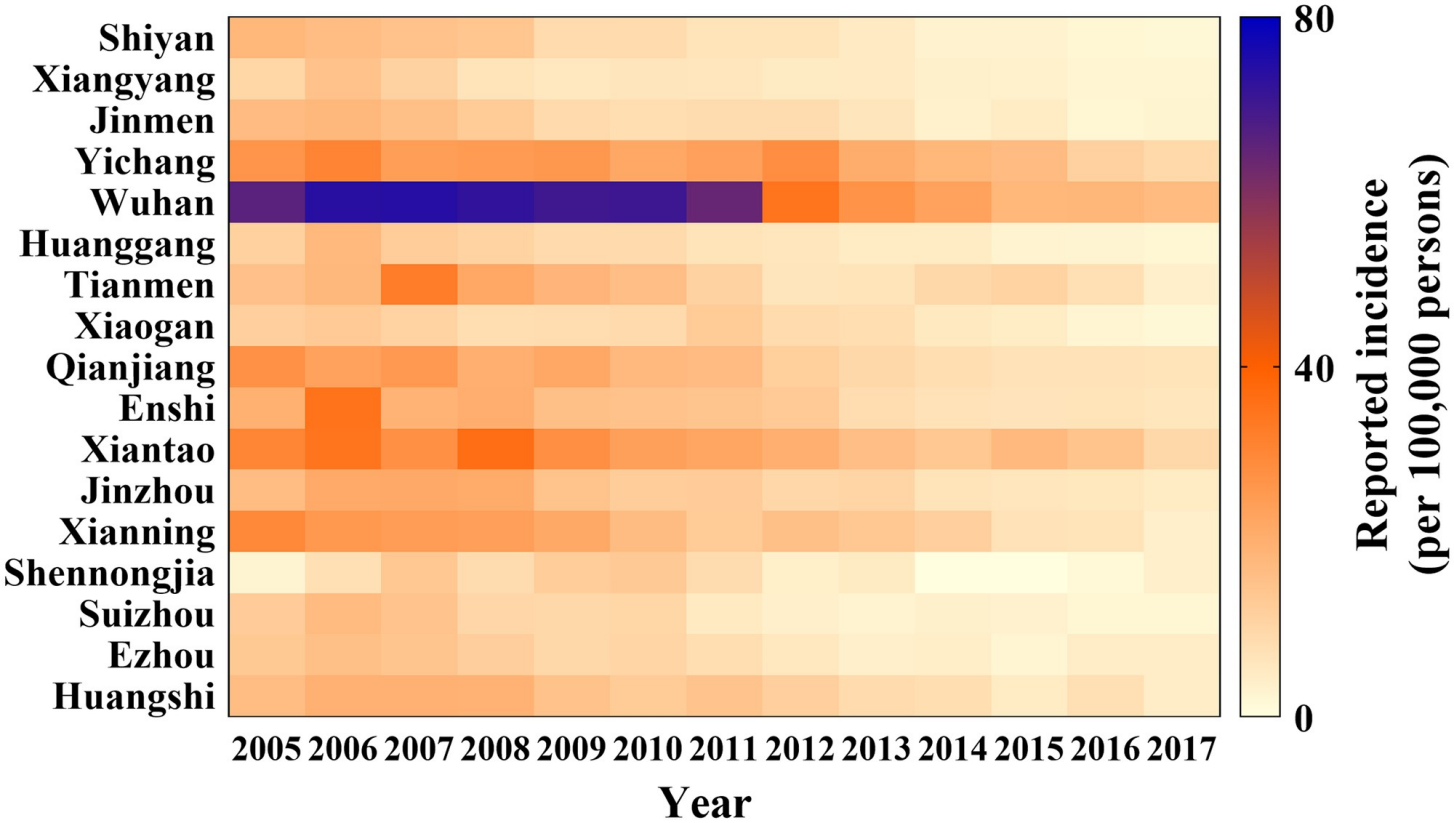
The reported incidence of shigellosis of all cities in Hubei Province from 2005 to 2017.

The incidence rate of shigellosis in all cities in Hubei Province gradually decreased year-on-year, except in 2005 (Fig 3). In Hubei Province, Wuhan City had the highest incidence rate in 2007 (73.96/100,000 persons), which decreased to 17.07/100,000 persons in 2017. From 2006 to 2017, incidence rate decreased from 35.22 to 6.10/100,000 persons, from 34.64 to 9.58/100,000 persons and from 30.76 to 9.51/100,000 persons in Enshi City, Xiantao City, and Yichang City, respectively.

**Fig 4:**
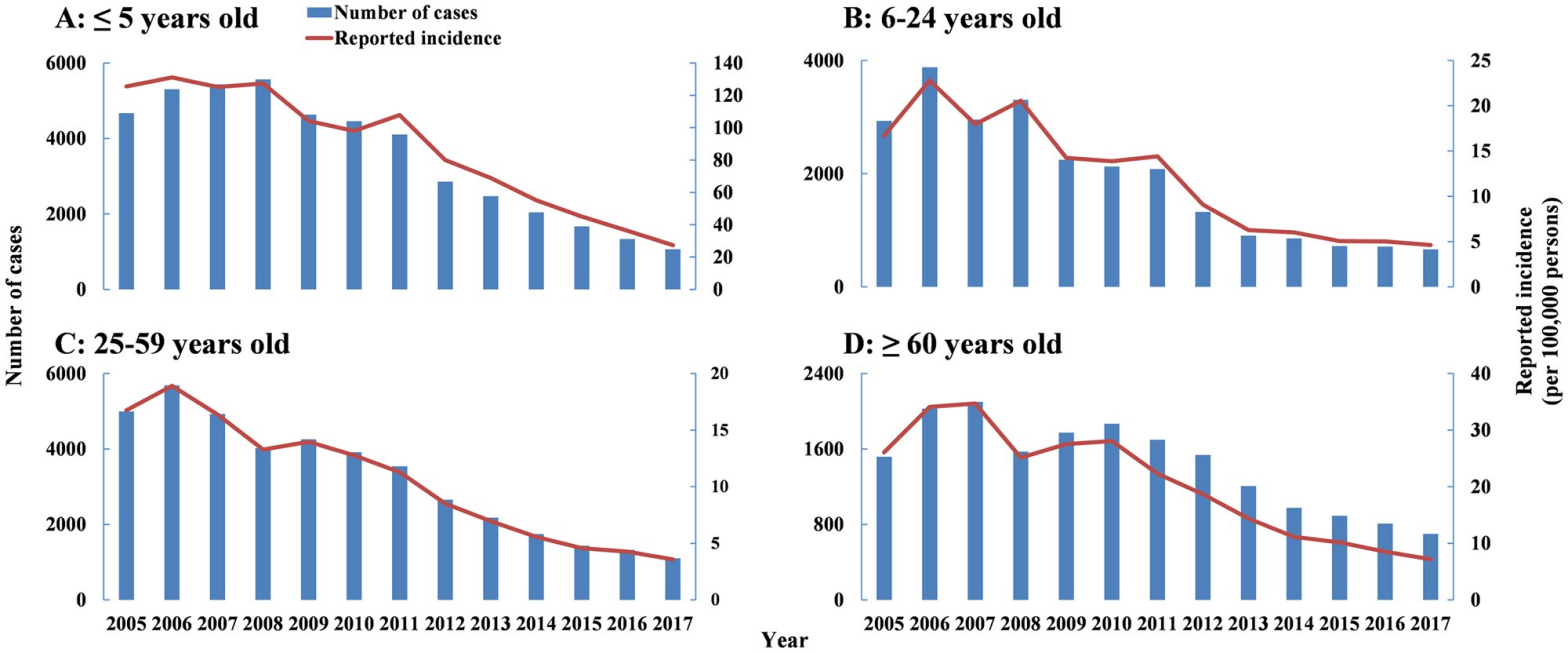
Reported cases and incidence of shigellosis in different age groups from 2005 to 2017 in Hubei Province. A) ≤ 5 years old. B) 6–24 years old. C) 25–59 years old. D) ≥ 60 years old.

From 2005 to 2017, 130,768 shigellosis cases (≤ 5 years: 45,601 cases; 6–24 years: 24,704 cases; 25–59 years: 41,791 cases; ≥ 60 years: 18,672 cases) were reported in Hubei Province (Fig 4). The incidence in all age groups decreased from 2005 to 2017.

### Curve fitting

**Fig 5:**
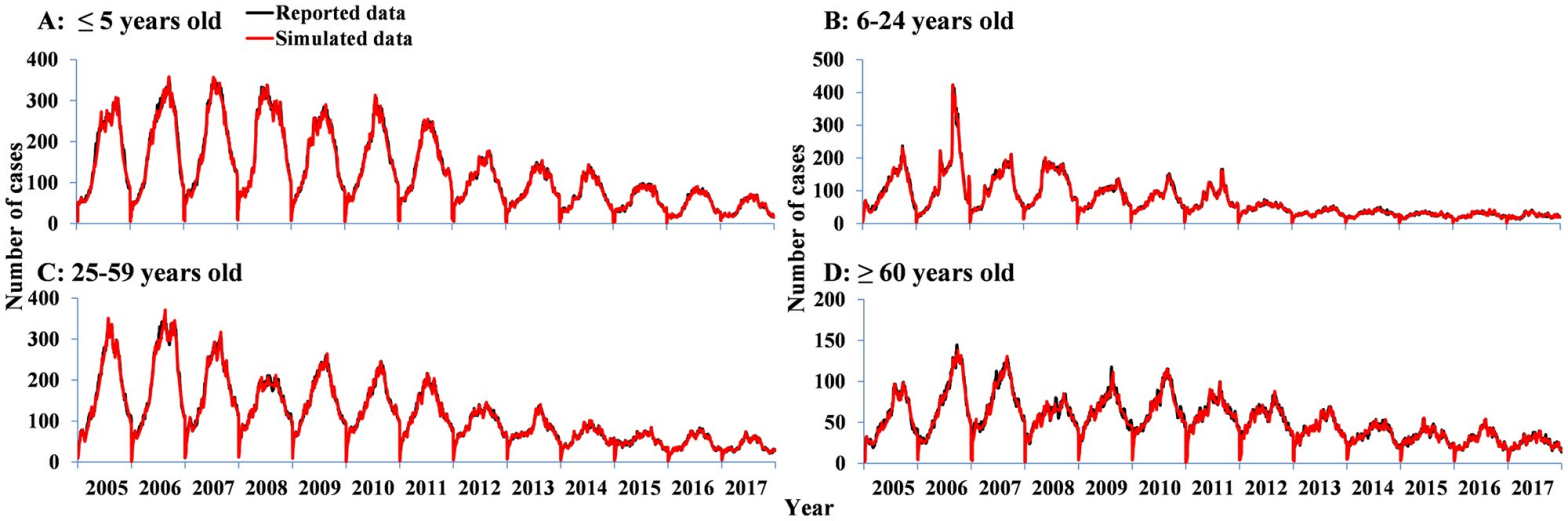
Curve fitting of model to reported data in different age groups from 2005 to 2017 in Hubei. A) ≤ 5 years old. B) 6–24 years old. C) 25–59 years old. D) ≥ 60 years old.

The age-specific SEIAR model fitted the reported data well in all of age groups (Fig 5). The *R*^*2*^ of the model by age group and year is shown in Table 3. The model simulated for shigellosis in 2007, 2010, 2013 and 2016 (see S1 Table) fitted the data well.

**Table 3:** *R*^*2*^ of model and reported cases in different age groups from 2005 to 2017 in Hubei Province, China.

Year	N	≤ 5		6–24		25–59		≥ 60	
		*R*^2^	*P*	*R*^2^	*P*	*R*^2^	*P*	*R*^2^	*P*
2005	365	0.986	<0.001	0.981	<0.001	0.992	<0.001	0.977	<0.001
2006	365	0.992	<0.001	0.994	<0.001	0.991	<0.001	0.991	<0.001
2007	365	0.993	<0.001	0.987	<0.001	0.991	<0.001	0.969	<0.001
2008	366	0.994	<0.001	0.990	<0.001	0.984	<0.001	0.926	<0.001
2009	365	0.993	<0.001	0.979	<0.001	0.992	<0.001	0.960	<0.001
2010	365	0.993	<0.001	0.983	<0.001	0.992	<0.001	0.978	<0.001
2011	365	0.989	<0.001	0.985	<0.001	0.990	<0.001	0.966	<0.001
2012	366	0.974	<0.001	0.959	<0.001	0.985	<0.001	0.946	<0.001
2013	365	0.989	<0.001	0.947	<0.001	0.979	<0.001	0.959	<0.001
2014	365	0.986	<0.001	0.938	<0.001	0.982	<0.001	0.953	<0.001
2015	365	0.979	<0.001	0.904	<0.001	0.955	<0.001	0.934	<0.001
2016	366	0.991	<0.001	0.953	<0.001	0.983	<0.001	0.967	<0.001
2017	365	0.986	<0.001	0.918	<0.001	0.982	<0.001	0.932	<0.001

### The transmissibility of shigellosis among different age groups

**Fig 6:**
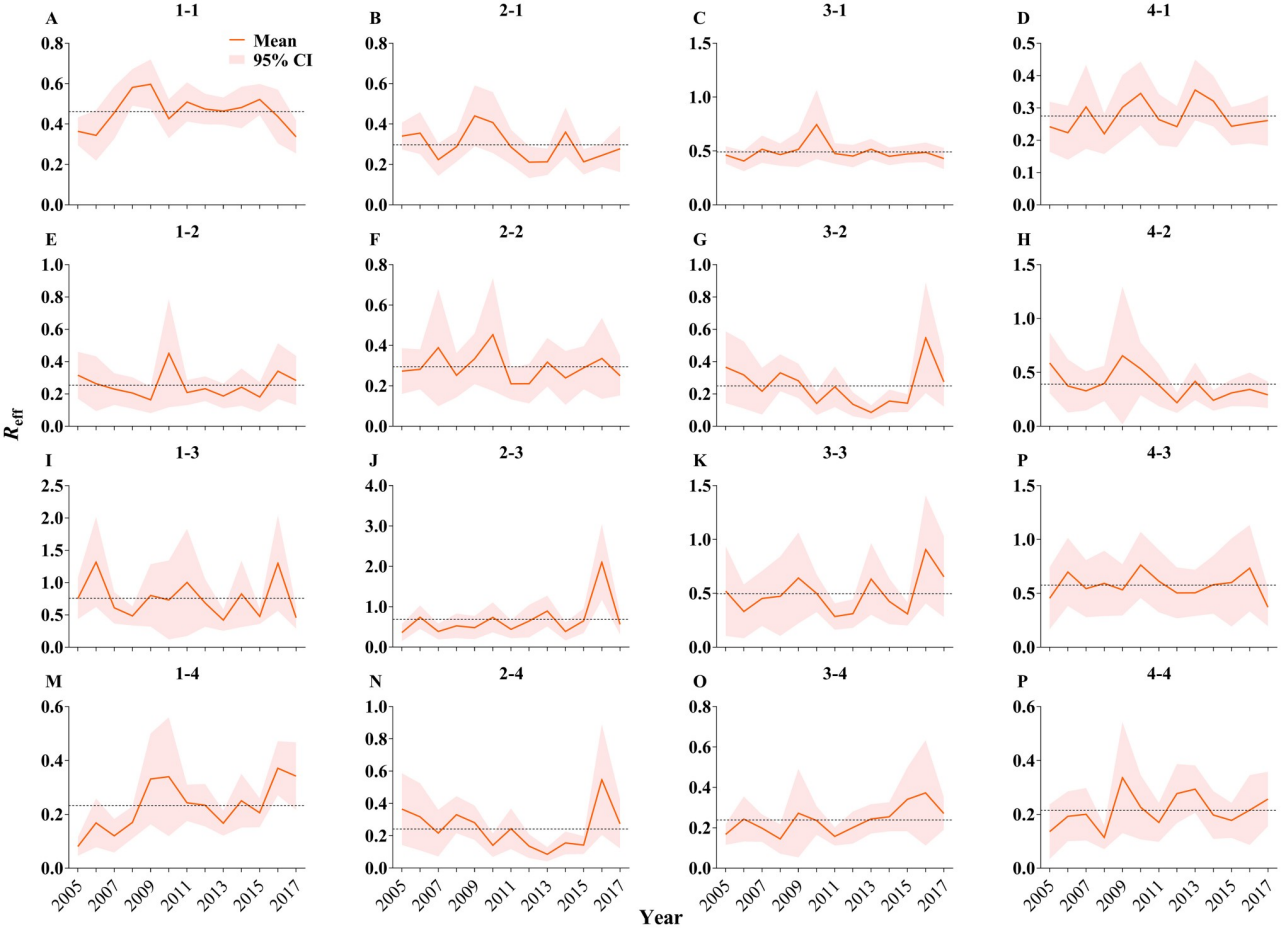
The *R*_eff_ trend of each transmission route from 2005 to 2017. The 1 to 4 in each subgraph represent age group ≤ 5 years old, 6–24 years old, 25–59 years old, and ≥ 60 years old, respectively. The black dot line indicates the mean value of *R*_eff_. –means transmission route, such as 1–4 means transmission from ≤ 5 years old to ≥ 60 years old.

The highest transmission interaction was between the ≤ 5 years and 25–59 years. The transmissibility in the ≤ 5 years was mainly within the group (mean *R*_eff_: 0.46, 95% CI: 0.36–0.56) (Fig 6A) and the 25–59 years (mean *R*_eff_: 0.49, 95% CI: 0.37–0.61) (Fig 6C). The transmission to the 25–59 years was mainly from the ≤ 5 years (mean *R*_eff_: 0.76, 95% CI: 0.34–1.17) (Fig 6I).

**Fig 7:**
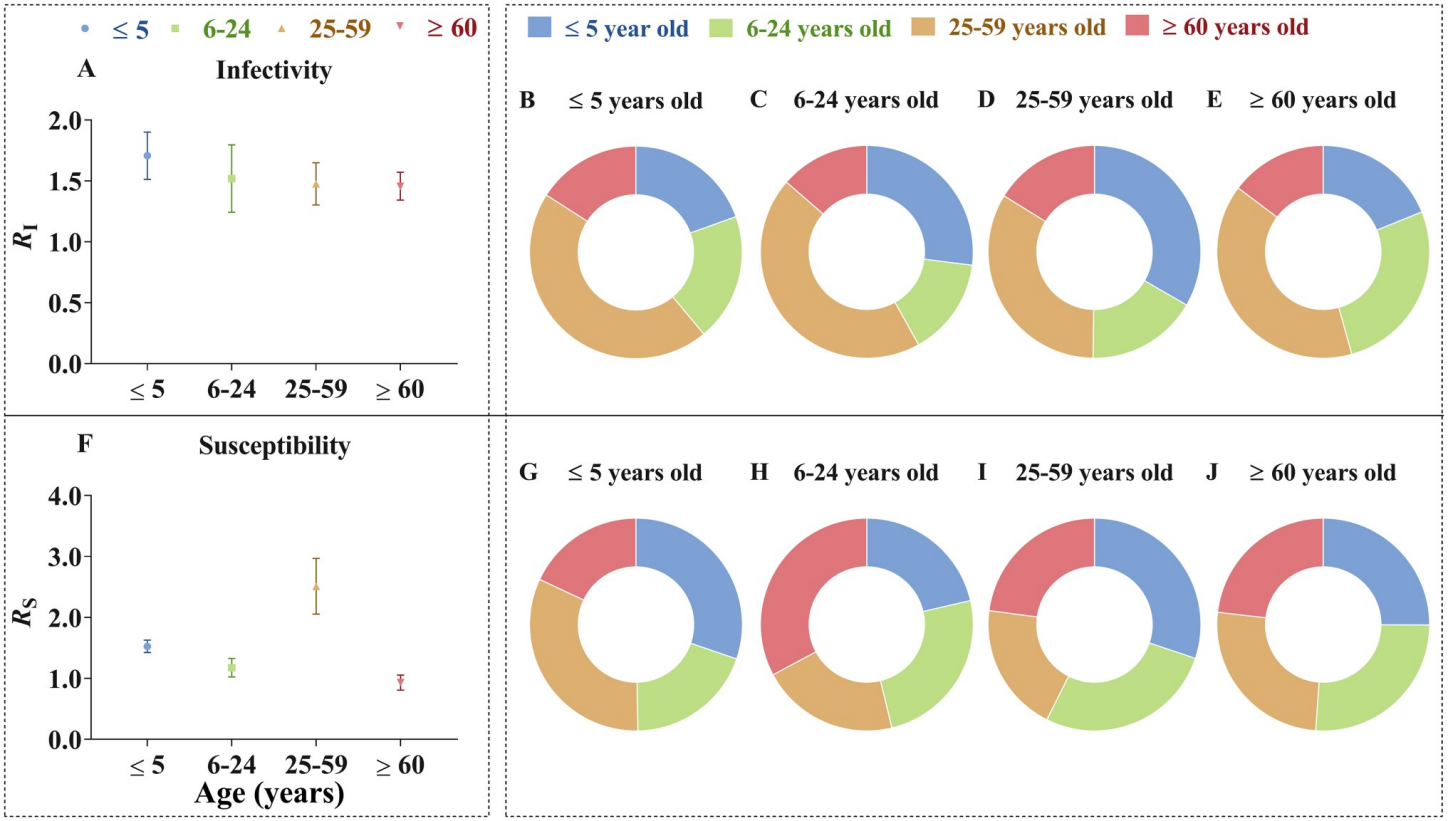
The infectivity, susceptibility and proportion of four age groups. The inside of the dashed line shows all subgraphs share one legend. *R*_I_ in Fig 7A indicates infectivity of each age group, such as infectivity of ≤ 5 years old (*R*_I-1_) equals to sum of *R*_eff-11_, *R*_eff-12_, *R*_eff-13_ and *R*_eff-14_. Fig 7B-7E respectively show the percentage of each age group in Fig 7A, such as there is a big percentage of infectivity from 25–59 years old to ≤ 5 years old in Fig 7B. *R*_S_ in Fig 7F is similar as description of Fig 7A, and Fig 7G-7J are similar as Fig 7B-7E.

Furthermore, the highest infectivity (Fig 7A) was in the ≤ 5 years (*R*_I_ = 1.71), which was most commonly transmitted to the 25–59 years (45.11%) (Fig 7B). The 25–59 years highest susceptibility (*R*_S_ = 2.51) (Fig 7F), and the most common source of transmission in this age group was from the ≤ 5 years (30.15%) (Fig 7I).

**Fig 8:**
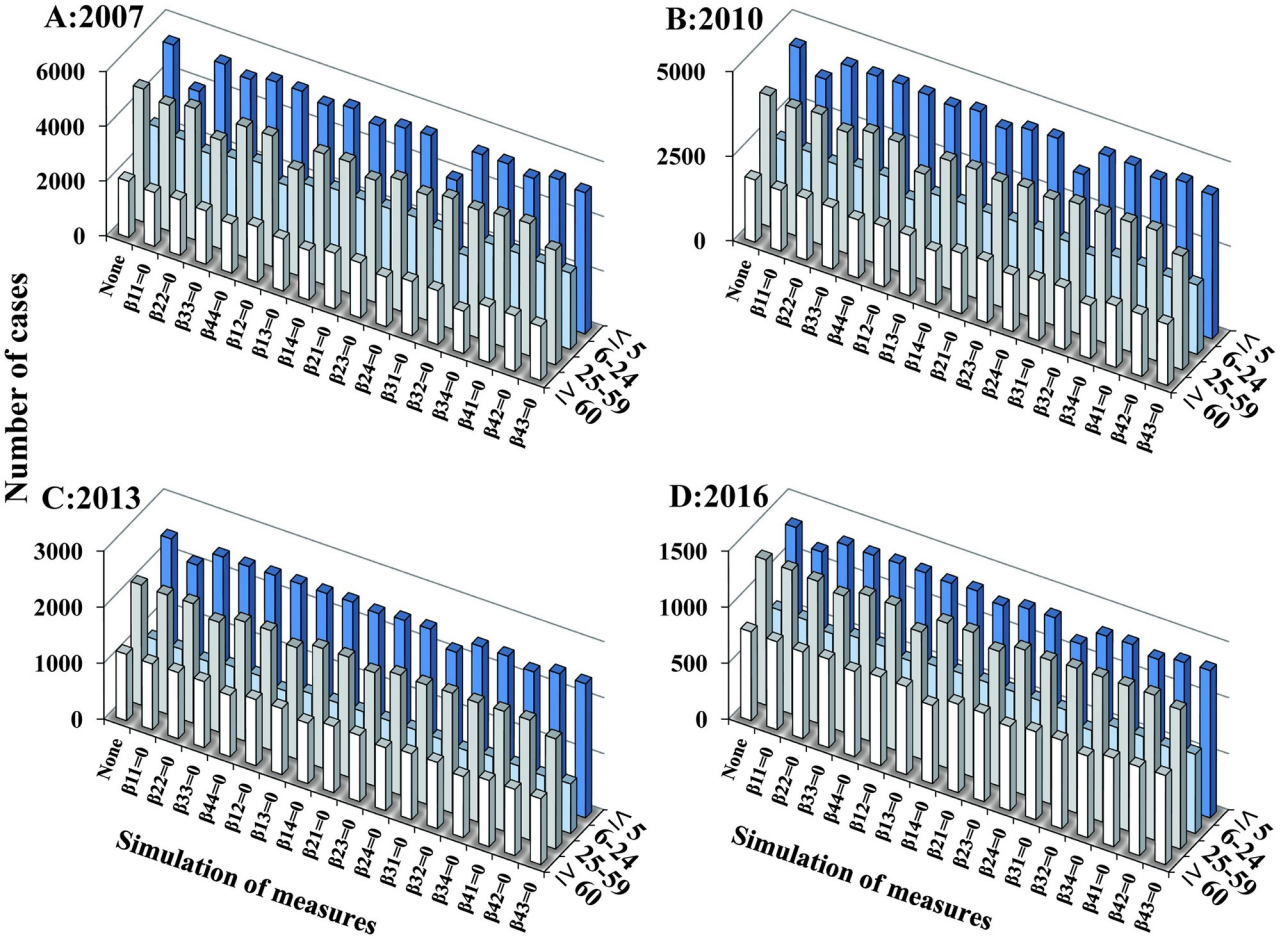
The “knock-out” simulation of scenarios I during the transmission in 4 stages. The bar shows the number of cases calculated without any intervention (control) or when cutting off just one transmission route (*β*_*ij*_ = 0, *i* and *j* respectively equal to 1, 2, 3 and 4). A) 2007. B) 2010. C) 2013. D) 2016.

The “knock-out” simulation produced similar *R*_eff_ results. In 2007 (Fig 8A), the number of cases in the ≤ 5 years could be reduced by 1,371 by cutting off transmission within the group (*β*_11_ = 0), and it could be reduced by 1,355 by cutting off transmission from the 25–59 years (*β*_31_ = 0). The number of cases in 25–59 years could be reduced by 993 by cutting off transmission from the ≤ 5 years (*β*_13_ = 0), and it could be reduced by 883 by cutting off transmission within the group (*β*_33_ = 0). In the simulation, cutting of transmission in the ≤ 5 years from within the group, or from the 25–59 years (*β*_11_ = 0 or *β*_31_ = 0, respectively), led to a large reduction in the number of cases in the ≤ 5 years in 2010, 2013, and 2016 (Fig 8). Furthermore, we obtained the greatest reduction in the number of cases in the ≤ 5 years when transmission was blocked both within the group and from the 25–59 years (both *β*_11_ = 0 and *β*_31_ = 0) (S4 Fig). The reduction in transmission increased as transmission was blocked within and between more groups (S5 Fig).

**Fig 9:**
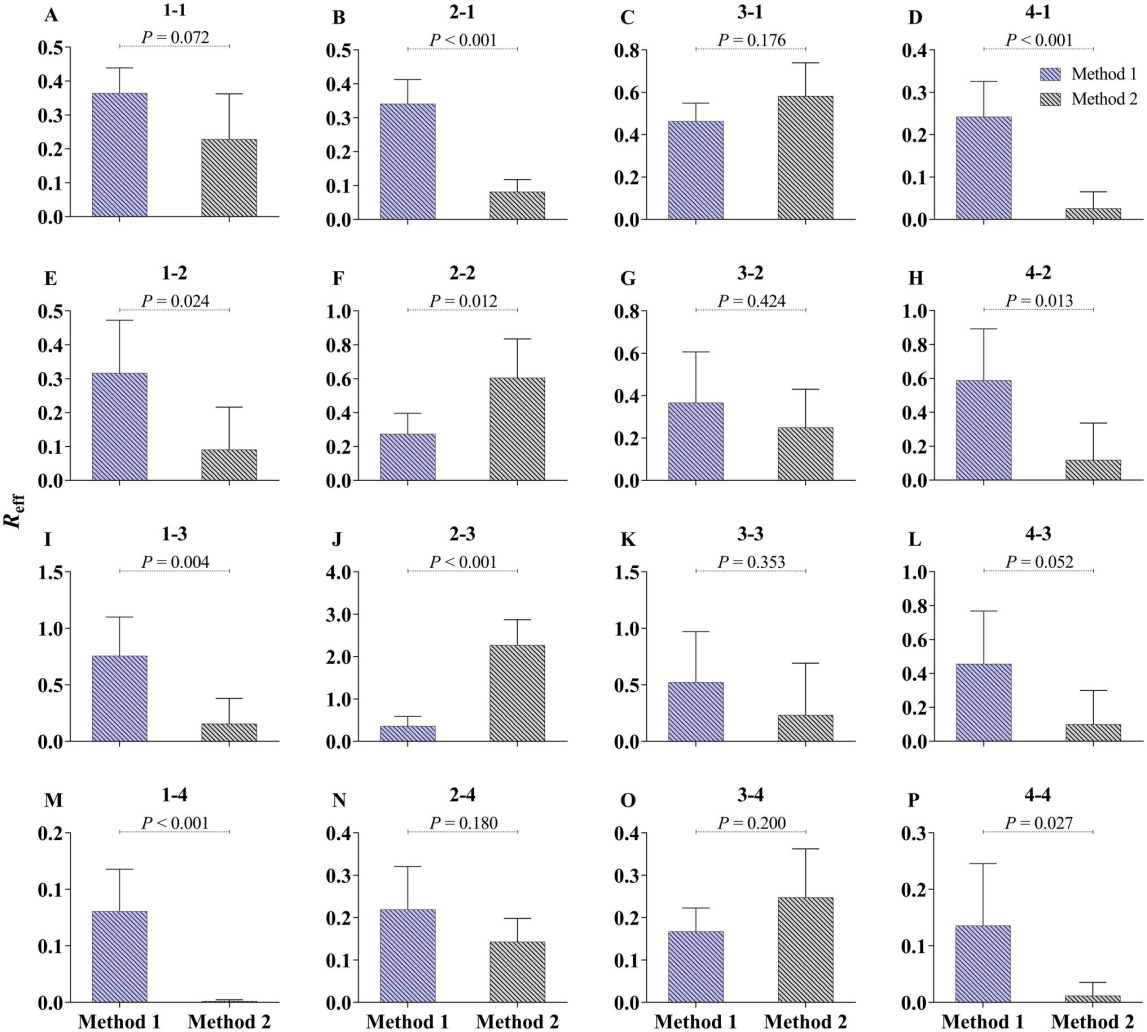
The comparison between method 1 and method 2. Method 1 is setting the initial values of *S* = total population of each year, *E* = 0, *I* = value at the end of the previous parts, *A* = 0, and *R* = 0; Method 2 is setting the initial values of *S*, *E*, *I*, *A* and *R* are same as the value at the end of the previous parts. The 1 to 4 in each subgraph represent age group ≤ 5 years old, 6–24 years old, 25–59 years old, and ≥ 60 years old, respectively.

There were some differences in the results of the two methods of curve fitting (Fig 9) but these differences were not significant in the seven transmission routes evaluated, including transmission within the ≤ 5 years (*P* = 0.072); from the 25–59 years to the ≤ 5 years (*P* = 0.176); from the 25–59 years to the 6–24 years (*P* = 0.424); within the 25–59 years (*P* = 0.353); from the ≥ 60 years to the 25–59 years (*P* = 0.052); from the 6–24 years to the ≥ 60 years (*P* = 0.180); or from the 25–59 years to the ≥ 60 years (*P* = 0.200).

### Reinfection analysis

**Fig 10:**
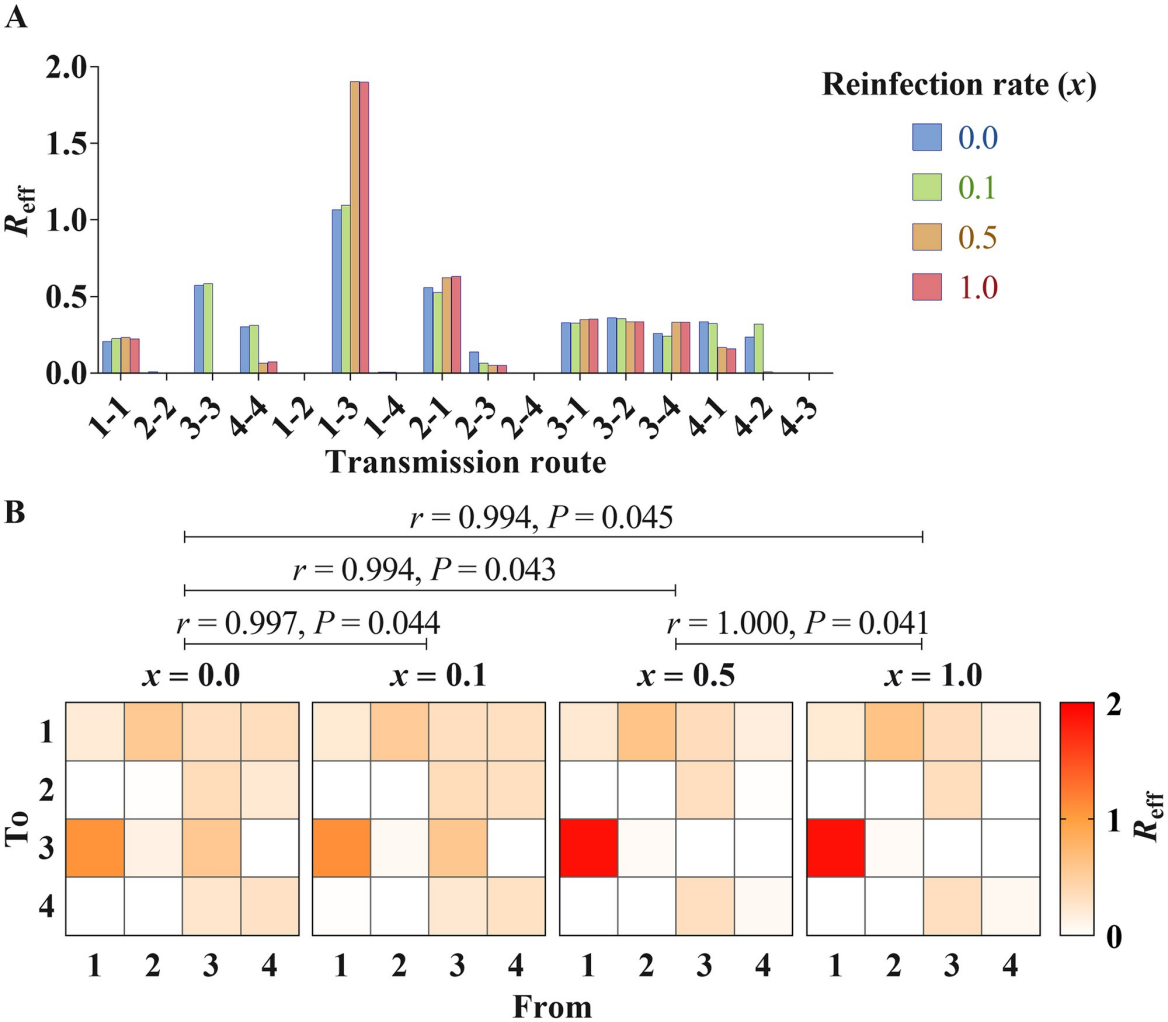
The influence analysis of reinfection of shigellosis in 2005. A) The different *R*_eff_ values from re-calibrating curve when setting *x* to 0.0, 0.1, 0.5 and 1.0, respectively; 1 to 4 represent age group ≤ 5 years old, 6–24 years old, 25–59 years old, and ≥ 60 years old, respectively. B) Mantel statistic r (*r*) and *P* value from Mantel Test of two matrices; matrix means transmission from one age group to another.

We obtained a similar result when setting *x* to 0 and 0.1, respectively, and when setting *x* to 0.5 and 1.0, respectively (Fig 10A). Although the four matrices are highly correlated, we have observed a little difference between *x* = 0.0 and *x* = 0.5 (Fig 10B).

### Sensitivity analysis

**Fig 11:**
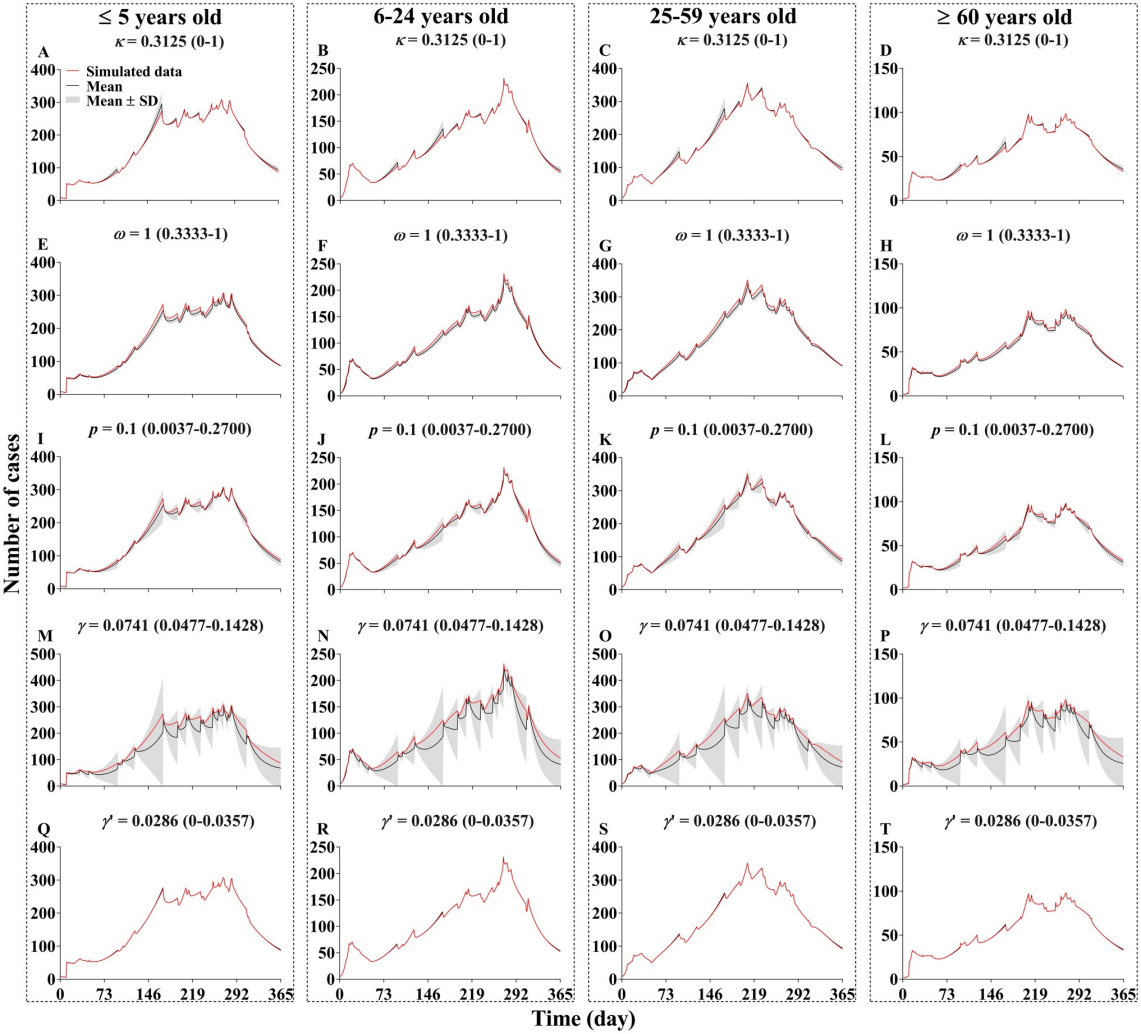
The sensitivity analysis of five parameters *κ*, *ω*, *p*, *γ*, and *γ*’. The red line shows the simulation result of the actual value in our model, and the black line and shadow gray band indicate the mean and standard deviation reserved after simulating 1000 times according to the value range of each parameter.

All the values of the parameters we set in the model were within the range of the simulated values of mean ± SD. The SEIAR model was not sensitive to parameters *κ*, *ω*, *p* and *γ*’, but had a high sensitivity for the parameter *γ* (Fig 11).

## Discussion

This study explored the person-to-person transmission mechanism of shigellosis among different age groups. The transmissibility, including infectivity and susceptibility, was calculated and compared. Our study provides guidance for controlling the person-to-person transmission of shigellosis.

### Validity of the model

In 2014, Tianmu et al. [17] verified the effectiveness of the SEIARW model with an outbreak dataset built by the Changsha CDC. After finding no contribution in the water/food-person route, a sex-specific SEIAR model was used to compare the transmission by sex [19]. Based on this, an age-specific SEIAR model was developed to quantify the different transmissibility in different age groups. The model had a good fit with the reported data in all four age groups. The most sensitive parameter was the infectious period, which suggests that it would be useful to collect data directly, rather than make assumptions based on the literature. There were some differences in the results of the simulations using the two methods of curve fitting, and so the fitting method should be explored when setting the initial values.

### Epidemiological characteristics

Shigellosis is the second most common cause of diarrheal death globally [2]. Most deaths occur in south Asia and sub-Saharan Africa [1]. In China, shigellosis causes a high disease burden in children under the age of 5 years [25,30,31]. This is similar to our results which showed the incidence rate in individuals aged ≤ 5 years was higher than that in the other age groups. The different age groups have different lifestyles, and the contact rate among children is higher than among adults, which leads to a high incidence in children aged ≤ 5 years old. The annual incidence in Wuhan City was higher than that in other cities from 2005 to 2017. Another study found that the incidence of shigellosis in Wuhan City remained high from 2006 to 2011 [32]. The main reason may be the high population density of Wuhan and the higher contact rate than in other cities. According to the Hubei Statistical Yearbook in 2018, the population of Wuhan City accounted for 18.46% of the population of Hubei Province. The recent reduction in shigellosis incidence is related to improvements of the water and sanitation infrastructure [12]. According to several studies [1,18,19], the transmission route of shigellosis has shifted from water/food-to-person to person-to-person spread. Therefore, it has become important to explore the transmissibility in different population groups, defined by factors such as sex, age, and occupation.

### Transmissibility of shigellosis in different age groups

Shigellosis causes a disease with high morbidity and mortality globally, disproportionately affecting young children in middle-and low-income countries [1]. Our findings suggest that shigellosis has high infectivity in children aged ≤ 5 years. Preschool children include children living at home, but in China preschool children and their caregivers tend to gather in larger groups. There is a direct correlation between shigellosis incidence and hygiene behaviors, such as handwashing [11]. Preschool children have a relatively high contact frequency with adults, but a poorer hand-hygiene behavior than adults. The highest incidence in children under 5 years old was caused by transmission within the age group, while the most common source of infection from outside the age group was from individuals in the 25–59 years age group. The highest susceptibility was found in individuals aged 25–59 years, which may be related to the tendency of individuals in this age group to take care of others at home. Therefore, the interaction of transmission was mainly among the ≤ 5 years age group and between the ≤ 5 years age group and the 25–59 years age group years. A study found that a significant proportion of mothers of children under five had poor handwashing practice in Debark town, northwest Ethiopia [33]. Handwashing is considered to be the most effective intervention for reducing the incidence of diarrhea. This may also be one of the reasons why individuals aged 25–59 years old had a high susceptibility to shigellosis. However, our previous study which used a sex-age-specific SEIAR model found that the most important transmission was mainly from older female (≥ 60 years old) to male children (≤ 5 years) [19]. The transmission features among different age and sex groups should be further explored.

We used the “knock-out” simulation that we developed to quantify the contribution of different transmission routes [19,21]. As with the estimate of the sources of transmission, we found that the transmission interaction was greatest among the ≤ 5 years age group and between the ≤ 5 years and the 25–59 years age groups. Furthermore, there was a great effect in reducing the incidence in the ≤ 5 years age group by cutting off the transmission routes among the group and from the 25–59 years age group. This finding suggests that the epidemic of shigellosis in children aged ≤ 5 years could be controlled by first applying interventions to these age groups. The isolation and treatment of shigellosis cases in individuals aged ≤ 5 years and 25–59 years may be an effective control strategy.

The “knock-out” simulation also showed the effectiveness of blocking transmission in these age groups. Our previous study showed that decreasing the infectious period and case isolation contained the transmission of shigellosis [34]. The above interventions could also be applied to control shigellosis in different age groups, but it is very important to apply optimized intervention measures. Using age-specific intervention strategies may be the most effective way to control of shigellosis. Control measures could include the following: a) enhancing the hygienic behaviors such as handwashing among children aged ≤ 5 years and adults aged 25–59 years; b) isolating and treating cases in children aged ≤ 5 years; and c) limiting the contact frequency among children aged ≤ 5 years old.

Although shigellosis may recur due to reinfection, reinfection did not affect our results if we assumed that the reinfection rate was ≤ 10%, but had a marked effect on the results if we assumed that the reinfection rate was > 50%; therefore, our data should be collected to obtain an estimate of the reinfection rate.

## Limitations

This study had some limitations. We ignored the impact of environmental factors (water and food) on shigellosis in this model. Most studies have indicated that *S*. *flexneri* (66.7%) and *S sonnei* (25%) are the two leading causes of endemic shigellosis in low- and middle-income countries [35,36]. However, the surveillance data for shigellosis did not include information on the types of *Shigella*. Furthermore, our study could not include the seasonality and ageing of the population in the dynamic process, because we employed a piecewise method of curve fitting. We need to strengthen the notification rate and determine the care-seeking behavior among individuals with shigellosis. Water/food transmission may still play an important role in transmission events that are often further propagated by person-to-person transmission [1], which means that the control framework should be adjusted to correspond to the specific transmission events. There might be differences between children and adolescents. However, we did not differentiate among children, adolescents, and adults aged > 18 years. Further studies are needed to explore the different patterns of transmission among children, adolescents, and adults.

## Conclusions

In Hubei Province, the transmission in children aged ≤ 5 years occurred among themselves, but was most commonly caused by transmission from individuals aged 25–59 years. The main transmission interaction was among children aged ≤ 5 years and between children aged ≤ 5 years and adults aged 25–59 years. Infectivity was highest among children aged ≤ 5 years and susceptibility was highest in adults aged 25–59 years. Intervention measures should thus be applied primarily to in these two age groups.

## Supporting information

S1 Fig Choice basis of 4 stages divided by shigellosis incidence from 2005 to 2017. The numbers in parentheses show the average reported incidence in each stage.
(TIF)

S2 Fig Flowchart of reinfection in age-specific SEIAR model of shigellosis. (TIF)

S3 Fig The division parts according to number of cases reported per day of Hubei Province in 2005. The red numbers on the dot line show that the data in 2005 was divided into 22 parts for curve fitting.
(TIF)

S4 Fig The “knock-out” simulation of scenarios II in combination of different routes transmitting to age ≤ 5 years in 2007. None represent without any intervention. *β*_I_ is setting *β*_11_ and *β*_21_ to 0; *β*_II_ is setting *β*_11_ and *β*_31_ to 0. *β*_III_ is setting *β*_11_ and *β*_41_ to 0. *β*_IV_ is setting *β*_21_ and *β*_31_ to 0. *β*_V_ is setting *β*_21_ and *β*_31_ to 0. *β*_VI_ is setting *β*_41_ and *β*_31_ to 0; *β*_VII_ is setting *β*_11_ and *β*_21_ to 0. *β*_VIII_ is setting *β*_11_, *β*_21_ and *β*_31_ to 0. *β*_IX_ is setting *β*_21_, *β*_31_ and *β*_41_ to 0. *β*_X_ is setting *β*_11_, *β*_21_, *β*_31_ and *β*_41_ to 0. The 1 to 4 represent age group ≤ 5 years old, 6–24 years old, 25–59 years old, ≥ 60 years old, respectively.
(TIF)

S5 Fig The “knock-out” simulation of scenarios III during the transmission in 4 stages. None represent without any intervention. *β*_I_ is setting *β*_11_, *β*_12_, *β*_33_ and *β*_34_ to 0. *β*_II_ is setting *β*_I_, *β*_31_, *β*_32_, *β*_13_ and *β*_44_ to 0. *β*_III_ is setting *β*_II_, *β*_21_, *β*_22_, *β*_43_ and *β*_14_ to 0. *β*_IV_ is setting *β*_III_, *β*_41_, *β*_42_, *β*_23_ and *β*_24_ to 0.
(TIF)

S1 Table The value of *β*_*ij*_ in each year and *R*^2^ in 4 stages in Hubei Province. (XLSX)
