# Supplementary figures and images for: Relative transmissibility of shigellosis among different age groups: A modeling study in Hubei Province, China
Source: PLoS Negl Trop Dis. 2021 Jun 10;15(6):e0009501. doi: 10.1371/journal.pntd.0009501 (PMC8219151; doi:10.1371/journal.pntd.0009501)

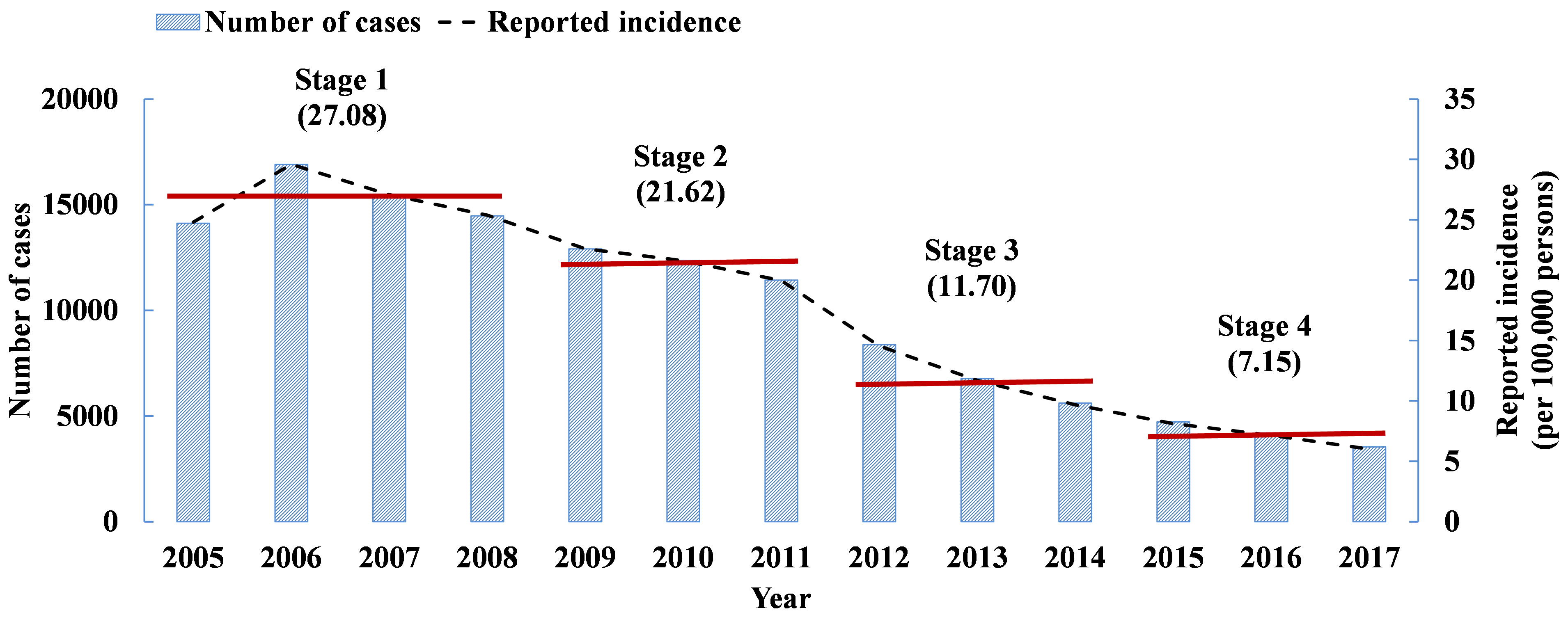

Supplement: S1 Fig — The numbers in parentheses show the average reported incidence in each stage. (TIF) [file pntd.0009501.s001.tif]

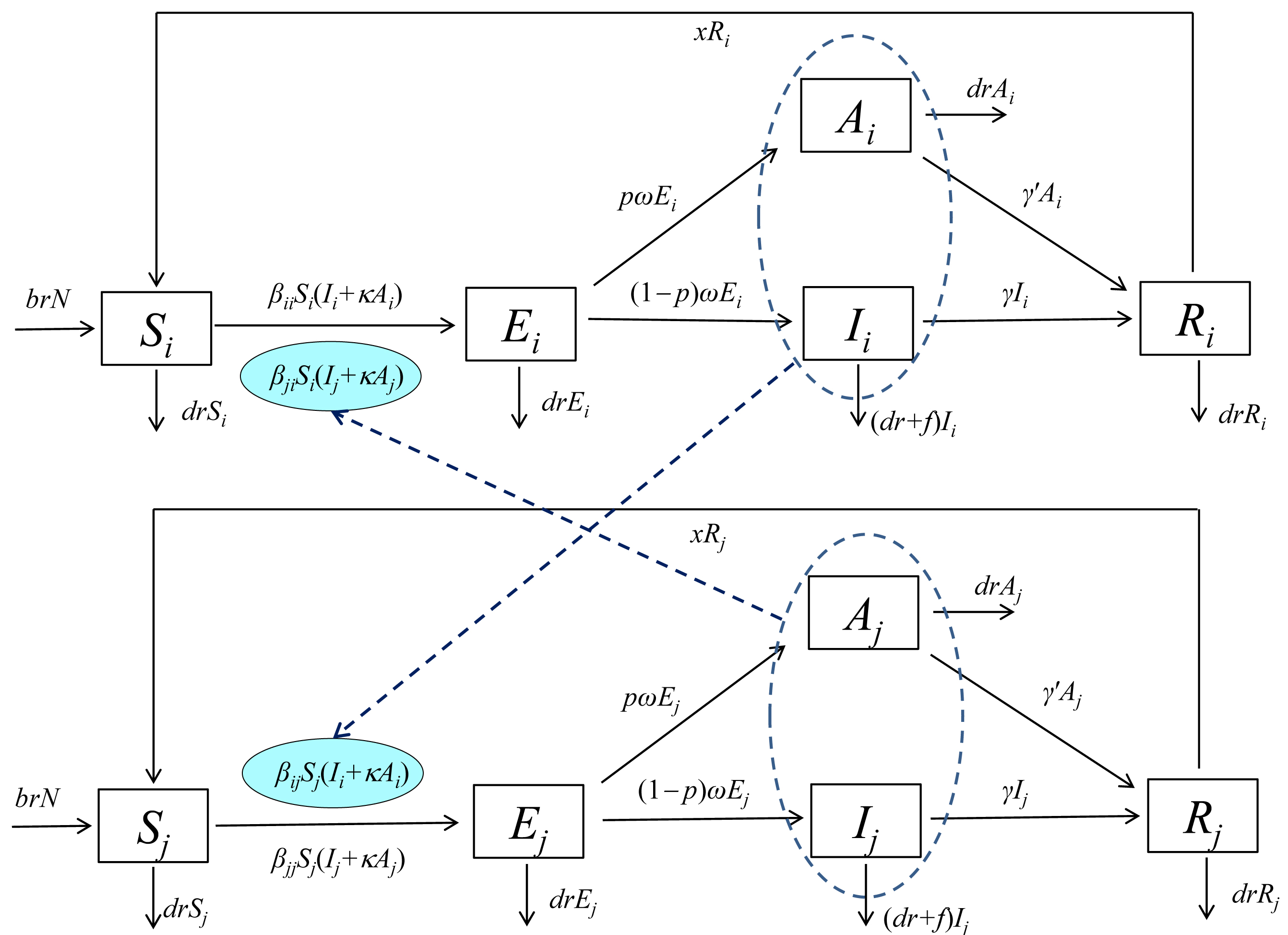

Supplement: S2 Fig — (TIF) [file pntd.0009501.s002.tif]

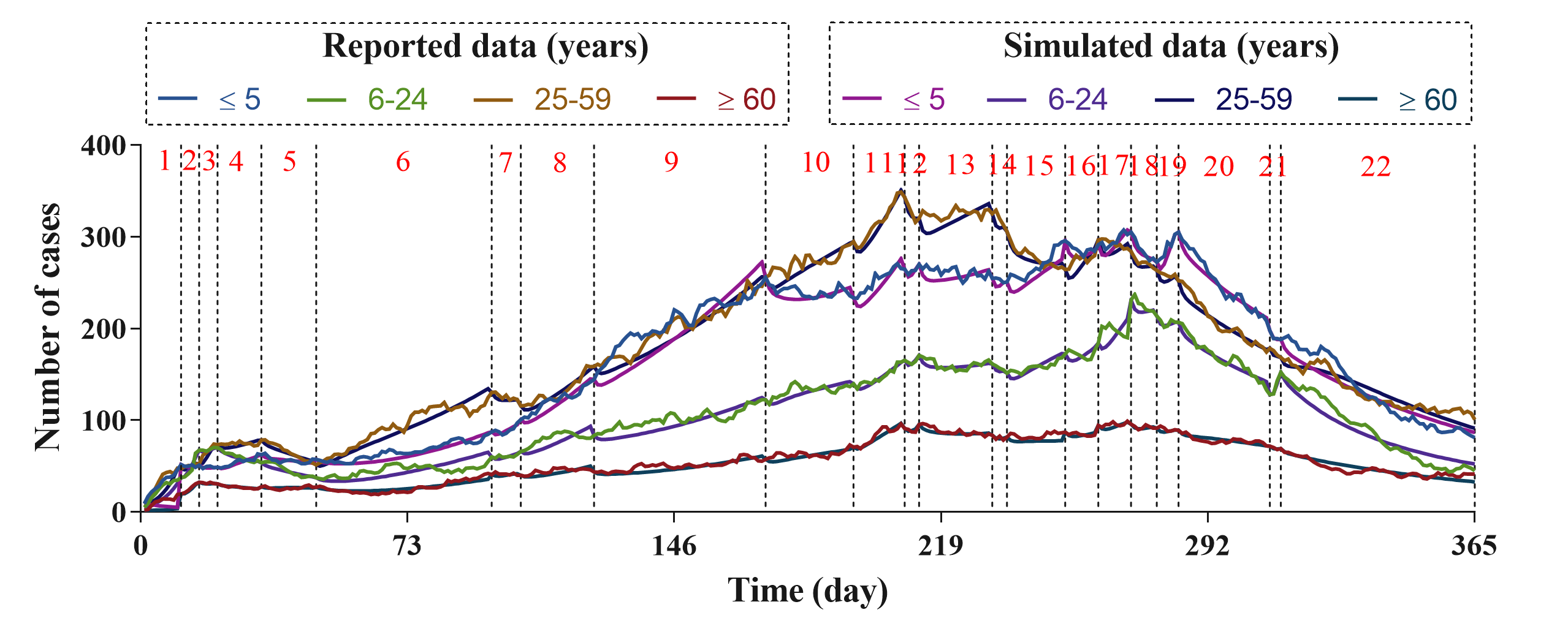

Supplement: S3 Fig — The red numbers on the dot line show that the data in 2005 was divided into 22 parts for curve fitting. (TIF) [file pntd.0009501.s003.tif]

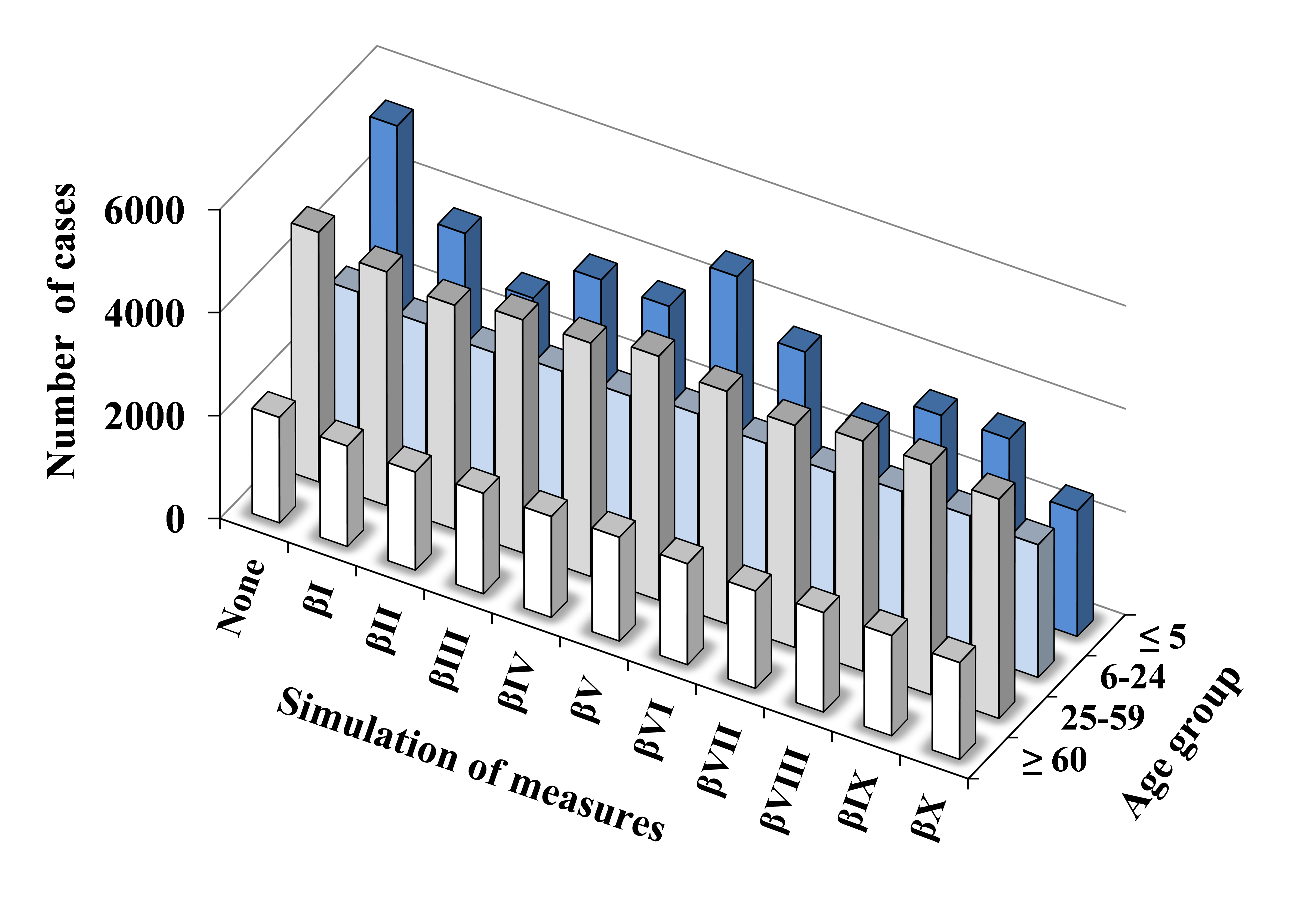

Supplement: S4 Fig — None represent without any intervention. βI is setting β11 and β21 to 0; βII is setting β11 and β31 to 0. βIII is setting β11 and β41 to 0. βIV is setting β21 and β31 to 0. βV is setting β21 and β31 to 0. βVI is setting β41 and β31 to 0; βVII is setting β11 and β21 to 0. βVIII is setting β11, β21 and β31 to 0. βIX is setting β21, β31 and β41 to 0. βX is setting β11, β21, β31 and β41 to 0. The 1 to 4 represent age group ≤ 5 years old, 6–24 years old, 25–59 years old, ≥ 60 years old, respectively. (TIF) [file pntd.0009501.s004.tif]

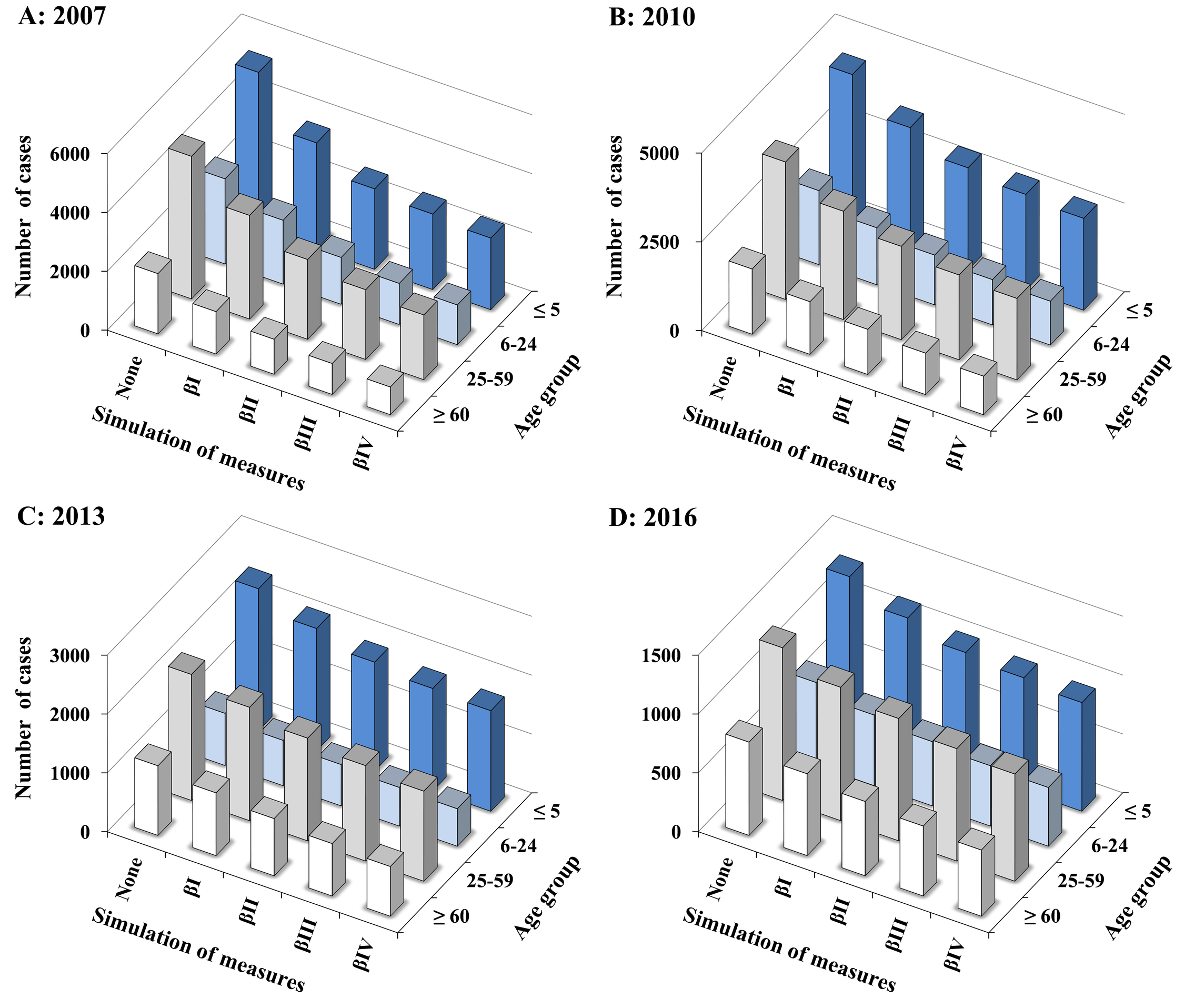

Supplement: S5 Fig — None represent without any intervention. βI is setting β11, β12, β33 and β34 to 0. βII is setting βI, β31, β32, β13 and β44 to 0. βIII is setting βII, β21, β22, β43 and β14 to 0. βIV is setting βIII, β41, β42, β23 and β24 to 0. (TIF) [file pntd.0009501.s005.tif]
